# Artery of Percheron infarction with persistent amnesia: a case report of bilateral paramedian thalamic syndrome

**DOI:** 10.1186/s12883-020-01949-0

**Published:** 2020-10-08

**Authors:** Hannah E. Snyder, Sheliza Ali, Joanna Sue, Ayse Unsal, Crystal Fong, Zhihui Deng

**Affiliations:** 1 Michael G. DeGroote School of Medicine, McMaster University, Hamilton, Canada; 2 Regional Rehabilitation Centre, Hamilton Health Sciences, Hamilton, Canada; 3 Department of Psychology, University of Victoria, Victoria, Canada; 4 Department of Psychiatry and Behavioural Neurosciences, McMaster University, Hamilton, Canada; 5 Department of Radiology, McMaster University, Hamilton, Canada; 6 Division of Physical Medicine and Rehabilitation, Department of Medicine, McMaster University, Hamilton, Canada

**Keywords:** Artery of Percheron, Paramedian thalamus, Neuropsychological assessment, Anterograde and retrograde amnesia

## Abstract

**Background:**

The artery of Percheron is an uncommon anatomic variant which supplies the bilateral paramedian thalami and rostral midbrain. While infarction of its vascular territory can result in a wide range of symptoms, paramedian thalamic syndrome is classically described as a triad of symptoms including vertical gaze disturbances, fluctuating level of consciousness, and amnesia. There is minimal evidence to date to characterize the long-term cognitive consequences of infarction of the artery of Percheron utilizing neuropsychological assessment.

**Case presentation:**

We describe a 40-year-old female patient initially presenting with dizziness, confusion and falls with unremarkable head CT scans. Subsequent MRI, more than 24 h after symptom onset, identified evidence of bilateral thalamic and rostral midbrain infarction. Neuropsychological testing was administered at 4 months post-stroke, with follow up testing at 1 year. The patient was found to have profound anterograde and retrograde amnesia, which did not change significantly over the first year of rehabilitation, and which was not easily identifiable in everyday encounters due to her relatively intact working memory and social skills.

**Conclusions:**

As early diagnosis of infarction of the artery of Percheron is challenging, patients have frequently missed the time window for acute management of ischemic stroke. Moreover, this case study highlights the need for further research in deciphering the role of the paramedian thalamus in memory and cognition, as well as the importance of standardized neuropsychological testing for the artery of Percheron stroke patients to identify safety and rehabilitation concerns that may be overlooked.

## Background

The artery of Percheron (AOP) is an uncommon anatomic variant first described by Gérard Percheron in 1973. It refers to a single arterial trunk originating from one of the proximal posterior cerebral arteries to supply bilateral paramedian thalami and rostral midbrain [1]. The AOP is thought to occur in up to 33% of the population, with a majority of individuals having independent hemispheric vascularization of these structures [2]. Infarction of this artery is often characterized by a triad of symptoms including altered level of consciousness, memory deficits, and supranuclear and vertical gaze palsies [3]. Rarer clinical presentations have been described, including patients presenting with severe bradycardia, parkinsonism and convulsive seizures [4–6]. Infarction within the vascular territory of the AOP is an important differential diagnosis to consider in an individual presenting to the Emergency Department (ED) with fluctuating level of consciousness (LOC), especially when considering that initial head computed tomography (CT) is typically normal [7]. Magnetic resonance imaging (MRI) is currently the gold standard for diagnosis of bilateral paramedian thalamic infarction.

On a macroscopic scale, the thalamus has been well-defined as a set of diencephalic nuclei which distributes motor and sensory information to and from the cortex. Meanwhile, the specific roles of individual nuclei within the paramedian thalamus, such as the dorsomedial and intralaminar nuclei, are still an area of controversy [1, 8]. The paramedian region is postulated to contribute to arousal, memory, personality and behavior based on various case reports describing sequelae such as reduced level of alertness, amnesia, apathy, and disinhibited behavior following bilateral infarction of this territory [3, 8]. Thus, neuropsychological assessment is an important component of initial testing of individuals with AOP infarction to determine degree of deficit in various cognitive domains. To our knowledge, only one case study of AOP infarction with neuropsychological assessment at two timepoints exists, and it describes a dramatic improvement of behaviour and cognition from 3 months to 1 year post-stroke [9]. Here, we present a case of a patient who similarly suffered bilateral paramedian thalamic infarction, but with a profoundly different outcome of severe amnestic syndrome and other cognitive deficits as identified by neuropsychological testing at 4 months and 1 year.

## Case presentation

Our patient was a 40-year-old right-handed woman with a previous history of recurrent deep vein thrombosis (DVT), reportedly related to oral contraceptive use and not managed medically. Otherwise, her past medical history was unremarkable for stroke risk factors. She awoke one morning complaining of dizziness and shortly thereafter began to act strangely, including missing a step while walking and getting into a low-impact collision while driving her son to work. She was alert and speaking clearly following this event, and her son suggested that she walk the short distance home. A couple of hours later, the patient was dysarthric and confused. She was subsequently found with evidence of trauma likely due to falls, including contusions on her feet, shin, and face for which she was not able to provide a history. Her initial Glasgow Coma Scale (GCS) was documented to be between 8 and 10 by Emergency Medical Services.

**Fig. 1:**
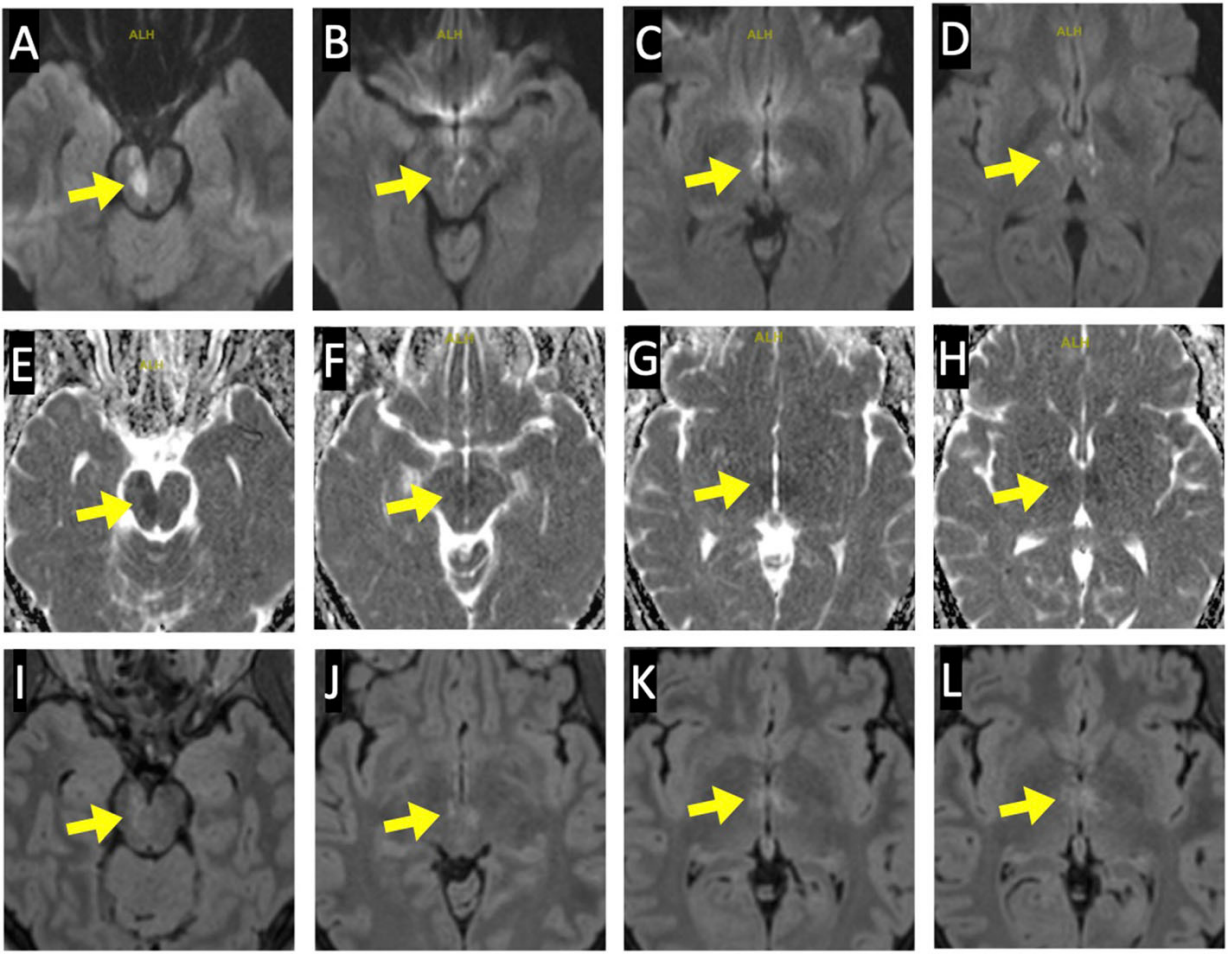
MRI findings of acute infarction in bilateral thalami and midbrain. Axial magnetic resonance images 1 day post-stroke demonstrating acute infarcts of the thalami and midbrain bilaterally (yellow arrow) as confirmed with increased signal on diffusion weighted imaging (**a-d**), corresponding signal drop out on apparent diffusion coefficient map (**e-h**), and hyperintensity on fluid attenuation inversion recovery sequences (**i-l**). For each row of images, axial slices are arranged caudally to rostrally

**Fig. 2:**
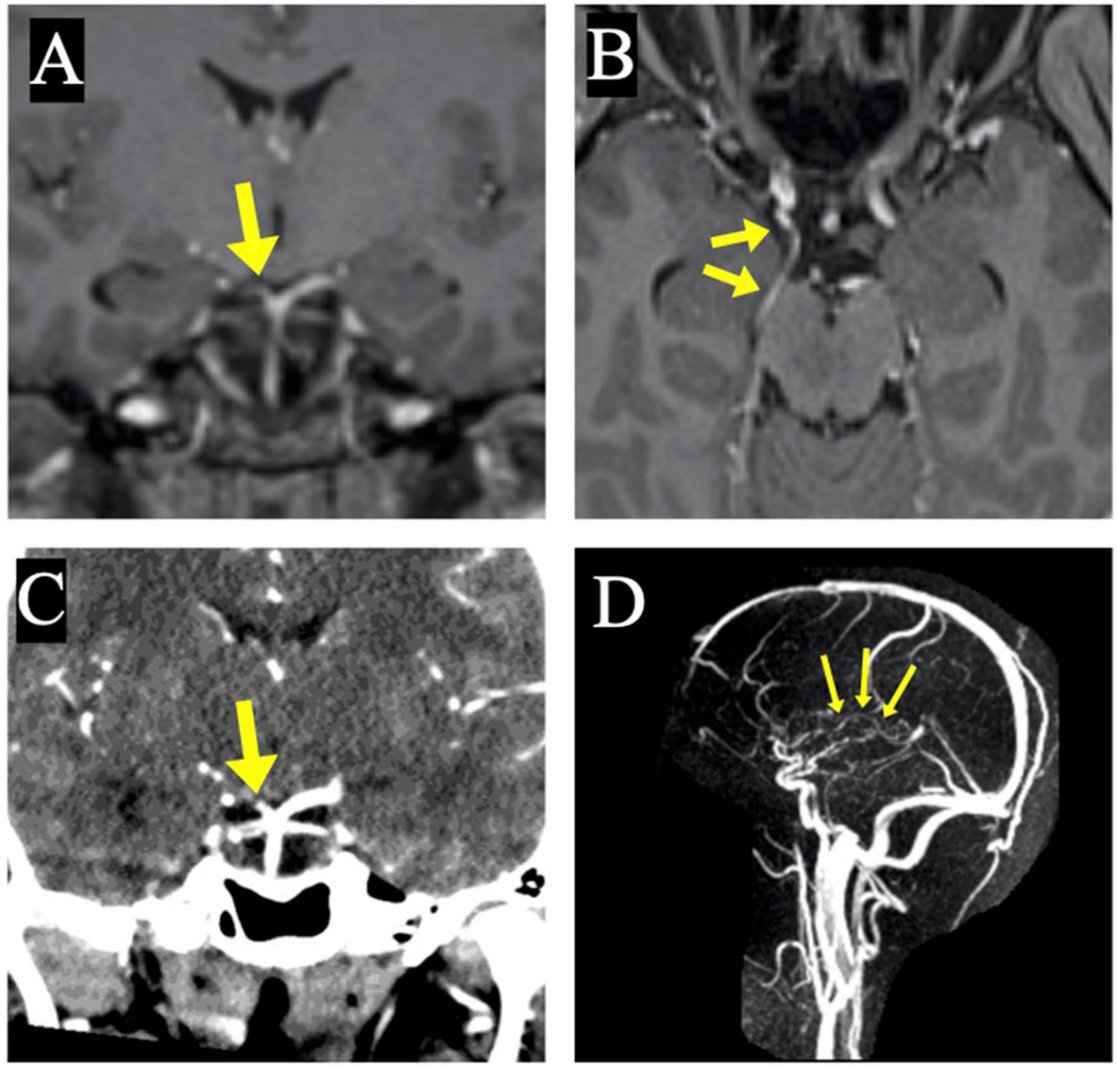
Angiographic findings suggestive of occlusion of right artery of Percheron. Contrast-enhanced magnetic resonance imaging (**a**) and CT angiogram (**c**) demonstrated normal opacification and diameter of the vertebrobasilar arterial trunk, excluding top of the basilar artery syndrome. There was slight asymmetry of the posterior cerebral arteries (PCAs) with relative narrowing of the right P1 segment compared to the left (yellow arrows in **a** and **c**), raising the possibility of incomplete P1 occlusion or stenosis and occlusion at the level of the perforating branches of the right PCA, namely artery of Percheron. However, the filling of the more distal part of the right PCA appeared normal, likely due to the contribution from a robust posterior communicating artery (yellow arrows in **b**). Magnetic resonance venogram (**d**) demonstrated normal opacification and filling of the internal cerebral veins (yellow arrows) and intracranial venous sinuses, excluding deep cerebral venous infarct

Upon arrival at the ED, our patient’s GCS deteriorated to 6 (motor 4, eyes 1, verbal 1) and she was temporarily intubated. Neurological exam was significant for subtle anisocoria with right pupil dilation, but both pupils were equally reactive to light and there was no gaze deviation. Initial medical workup including lumbar puncture and electroencephalography was unremarkable. CT head without contrast showed no acute intracranial abnormalities. A repeat CT head with angiogram performed the same day were also unremarkable. Finally, a brain MRI with angiogram the following day demonstrated evidence of acute infarcts in the bilateral thalami and midbrain (Fig. 1) with angiographic findings suggestive of occlusion of the right artery of Percheron (Fig. 2). Unfortunately, our patient was past the time window for thrombolysis. She was managed conservatively and then admitted to a local rehabilitation centre for post-stroke rehabilitation once medically stable. Investigations for stroke etiology, including coagulopathy screen, ultrasound for DVT, and transthoracic echocardiogram with contrast bubble study for intracardiac shunting, were unremarkable. A thorough vasculitis workup was not performed given the lack of evidence of brain angiitis on imaging or clinical signs of a systemic rheumatologic disease.

She was ultimately transferred to an Acquired Brain Injury (ABI) Rehabilitation Program 59 days following her initial presentation. Her admitting neurological examination revealed slightly reduced left-sided strength in both her upper and lower extremity without other focal neurological deficits. Meanwhile, significant cognitive impairment was identified, specifically involving her memory and executive functioning. Regarding functional status, she was using a wheelchair regularly for safety and cognitive reasons but was physically able to perform transfers and climb stairs with supervision, she was alert but not oriented, she was occasionally incontinent of urine, and she displayed lack of judgement and some impulsiveness. A trial of neurostimulants (amantadine up to 100 mg BID and methylphenidate up to 15 mg BID) aimed at improving her cognition did not lead to any functional benefit.

As an inpatient on the ABI unit, our patient underwent several days of standardized neuropsychological assessment at four months following her initial event. A battery of tests was administered over several sessions, which assessed her intellectual functioning, processing speed, attention, working memory, visuospatial function, memory, and executive functioning. She was noted to have a profound memory deficit, with performance in the lowest percentile on nearly all tests of immediate recall, delayed recall, and recognition (i.e. she was unable to recall or recognize either verbal or visual information after delays of approximately 20 to 30 min). While she tended to perform in the low average to average range on tests of working memory, she could not encode these memories into long-term storage. Moreover, consistent with a severe anterograde and retrograde amnesic syndrome, she self-reported an inability to form any new episodic memories or recall any events which occurred before her stroke, including the inability to recall memories of her childhood. While she also performed poorly on tests of executive function, it was difficult to determine the true extent of these deficits as her ability to perform these tasks and learn from feedback may have been confounded with her memory deficits. Importantly, her relatively intact basic attention, working memory, and social skills made it extremely difficult for those around her to determine the extent of her memory impairments. At 1 year follow-up, the patient’s neuropsychological assessment showed mild improvement in her visual memory functioning, but her other memory scores remained weak and consistent with her previous assessment (Table 1). Prior recommendations including 24-h supervision, repetition of information, provision of one-step instructions, and gradually increasing physical and mental activity were still being employed by her family with good tolerance of these strategies.

**Table 1:**
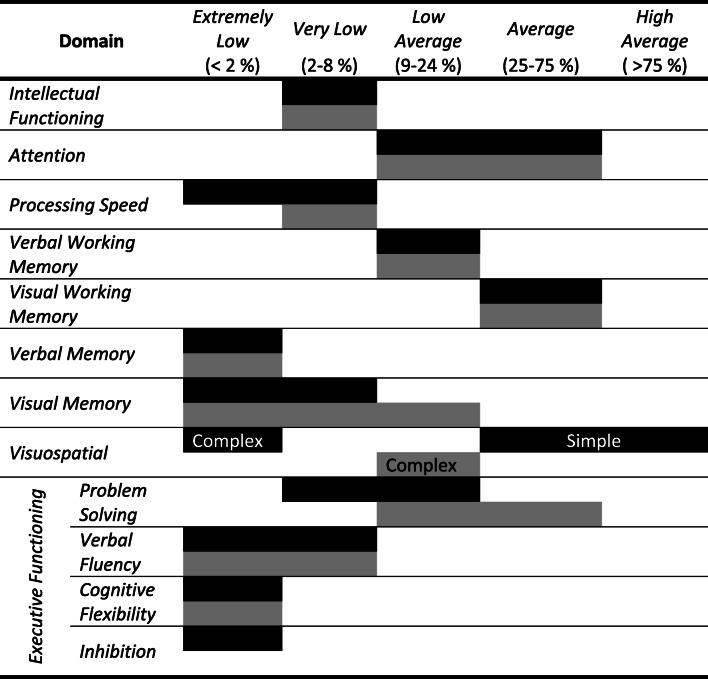
Neuropsychological profile post-injury at 4 months and 1 year post-injury

Neuropsychological testing at 4 months (black) and 1 year (grey) post-stroke. Performances that span across ranges represent variability within that domain. Simple visuospatial functioning and inhibition were tested at the 4 month timepoint only.

## Discussion and conclusions

AOP infarction is an often-delayed diagnosis due to various factors. First, the AOP is an uncommon anatomic variant and its stroke syndrome is not commonly encountered. Next, ischemia of this region can present in a variety of ways and is not limited to the classically described paramedian thalamic syndrome [3]. Finally, MRI is the gold standard for diagnosis of AOP infarction, while CT is routinely used as first-line imaging for standard stroke diagnosis [7]. In our case, MRI was performed more than 24 h after the patient’s initial symptom onset that identified restricted diffusion within the thalami and midbrain bilaterally. The early recognition of thalamic stroke syndromes as a cause of decreased LOC and coma is essential since hyperacute AOP infarction can be treated effectively with intravenous heparin and thrombolysis [10, 11].

This case emphasizes the need for neuropsychological assessments at various time points following AOP infarction to identify the true extent of cognitive impairment. While there have been cases where AOP infarction led to an amnestic presentation similar to Korsakoff syndrome with its pathognomonic symptom of confabulation, our patient’s profound anterograde and retrograde amnesia was not immediately obvious to caregivers due to her strong social skills and relatively intact working memory [12]. The extent of her deficit was only identified when she completed neuropsychological testing, which also allowed for appropriate care recommendations and prognosis to be delivered.

Although our patient did not show significant recovery, Krolak-Salmon et al. described a case of AOP infarction with memory impairment 1 month post-stroke, but complete recovery after 1 year [9]. While recovery trajectory has not been specifically analyzed in this population, a major study of anterior circulation ischemic strokes showed that functional outcome was best predicted by neurologic improvement at only 2 days post-infarction [13]. Accordingly, our patient’s outcome may be more representative of the typical stroke patients.

Regarding memory impairment, researchers have noted that patients with damage to the dorsomedial and intralaminar thalamic nuclei, which are impacted by AOP infarction, may present with similar memory difficulties as those with bilateral hippocampal damage [14]. On the other hand, prefrontal cortex (PFC) dysfunction is associated with executive function impairments [15] and some researchers have proposed that the memory deficits seen in AOP stroke are secondary to impairments in executive function, supported by the correlation between thalamic substructures and PFC [16]. Increasing evidence indicates interconnections between the PFC and the dorsomedial thalamus, although the mechanism of PFC-thalamic functional connectivity underlying higher cognitive function is not completely understood [17]. While there may be a relationship between our patient’s executive function and memory, given the severity of her memory deficits (i.e., anterograde and retrograde amnesic syndrome), it is unlikely that her executive abilities would fully account for all aspects of her memory impairment, such as her inability to form basic episodic memories.

Limitations of the current study include differences between administration of neuropsychological assessment at 4 months and 1 year after stroke. Particularly, the initial assessment was completed over several days, whereas follow-up was obtained in 1 day and included self-report measures of behaviour and personality as opposed to extensive clinical interviewing. Although testing was completed in different settings (inpatient versus outpatient) and there was some variation in the measures utilized, both were conducted by licensed neuropsychologists and similarly trained psychometrists. Future studies may take a more experimental approach by having a standard assessment protocol at each timepoint.

In closing, AOP infarction is an important differential diagnosis to consider when a patient presents with fluctuating consciousness characteristic of stroke but initial head CT is normal, since early diagnosis and management may significantly improve clinical outcome. We described a case of a 40-year-old female presenting with profound anterograde and retrograde amnesia who missed the time window for thrombolysis following AOP occlusion. Neuropsychological assessment at 4 months and 1 year post-stroke showed limited improvement over time. Importantly, her relatively intact social skills and working memory prevented appreciation of the true extent of her memory deficit in the absence of neuropsychological testing. Thus, this case study emphasizes the need for thorough neuropsychological testing to document areas of impairment and to identify strategies for rehabilitation and safety in AOP stroke patients. It also supports the role of the paramedian thalamic territory in executive function and memory as has been described by previous works, but additional research is needed to decipher the functions of specific thalamic nuclei, such as the dorsomedial nucleus, in moderating these cognitive domains.

## Data Availability

The authors declare that all the data are contained within the manuscript.

## Abbreviations

AOP: Artery of Percheron
ABI: Acquired brain injury
CT: Computed tomography
DVT: Deep vein thrombosis
ED: Emergency department
GCS: Glasgow Coma Scale
LOC: Level of consciousness
MRI: Magnetic resonance imaging
PFC: Prefrontal cortex
